# Production of TGF-β1 as a Mechanism for Defective Antigen-presenting Cell Function of Macrophages Generated *in vitro* with M-CSF

**DOI:** 10.4110/in.2009.9.1.27

**Published:** 2009-02-28

**Authors:** Jae Kwon Lee, Young-Ran Lee, Young-Hee Lee, Kyungjae Kim, Chong-Kil Lee

**Affiliations:** 1 School of Science Education (Biology), Chungbuk National University, Cheongju, Korea.; 2 College of Pharmacy, Chungbuk National University, Cheongju, Korea.; 3 College of Pharmacy, Sahmyook University, Seoul, Korea.

**Keywords:** macrophage, M-CSF, APC function, TGF-β1

## Abstract

**Background:**

Macrophages generated *in vitro* using macrophage-colony stimulating factor (M-CSF) and interleukin (IL)-6 from bone marrow cells (BM-Mp) are defective in antigen presenting cell (APC) function as shown by their ability to induce the proliferation of anti-CD3 mAb-primed syngeneic T cells. However, they do express major histocompatibility (MHC) class I and II molecules, accessory molecules and intracellular adhesion molecules. Here we demonstrate that the defective APC function of macrophages is mainly due to production of TGF-β1 by BM-Mp.

**Methods:**

Microarray analysis showed that TGF-β1 was highly expressed in BM-Mp, compared to a macrophage cell line, B6D, which exerted efficient APC function. Production of TGF-β1 by BM-Mp was confirmed by neutralization experiments of TGF-β1 as well as by real time-polymerase chain reaction (PCR).

**Results:**

Addition of anti-TGF-β1 monoclonal antibody to cultures of BM-Mp and anti-CD3 mAb-primed syngeneic T cells efficiently induced the proliferation of syngeneic T cells. Conversely, the APC function of B6D cells was almost completely suppressed by addition of TGF-β1. Quantitative real time-PCR analysis also confirmed the enhanced expression of TGF-β1 in BM-Mp.

**Conclusion:**

The defective APC function of macrophages generated *in vitro* with M-CSF and IL-6 was mainly due to the production of TGF-β1 by macrophages.

## INTRODUCTION

Macrophages can be generated from bone marrow (BM) progenitor cells by culturing in the presence of macrophage-colony stimulating factor (M-CSF). Although M-CSF alone can induce differentiation of BM progenitor cells into macrophages, the combination of M-CSF and interleukin-6 (IL-6) significantly enhances macrophage production from BM progenitors (1). The macrophages generated *in vitro* using M-CSF or M-CSF plus IL-6, however, appeared to be different from normal macrophages isolated from tissues. Although macrophages generated *in vitro* with M-CSF do express MHC class II molecules, numerous accessory molecules and intercellular adhesion molecules, they are defective in APC function (2-4). It has also been shown that some members of the macrophage family suppress antigen presentation by dendritic cells (DCs) (5), or induce T cell anergy (6), suppression (7) or apoptosis (8). Thus it has been suggested that differentiation along divergent pathways influences the APC function of various cell types. For instance, thymic macrophages are a specialized subset of macrophages that can phagocytose apoptotic cells very efficiently, but have poor APC functioning (9).

The APC function of mature macrophages may be affected by a variety of factors including the micro environmental condition of its development. As shown recently, acquisition of APC functioning of mature macrophages was significantly influenced during development by factors such as the presence of CC chemokines like Lkn-1, MIP-1α and RANTES together with M-CSF (10). The defective APC functioning of macrophages generated *in vitro* with M-CSF may also be linked to the intrinsic inability of the macrophages to produce cytokines such as IL-12 or to express an invariant (Ii) chain of class II MHC molecules (2,3).

In the present study, we demonstrate that macrophages generated *in vitro* with M-CSF and IL-6 from BM cells (BM-Mp) produce TGF-β1 in a quantity that is sufficient to suppress the proliferation of anti-CD3 mAb-primed syngeneic T cells. A macrophage cell line B6D, which exerts efficient APC function, produces much less TGF-β1 compared to BM-Mp. The present study, together with other studies which demonstrated that TGF-β1 inhibits the proliferation of T and B-lymphocytes, thymocytes and NK cells (11-15), indicates that the defective APC function of BM-Mp is mainly attributable to the production of TGF-β1 by BM-Mp.

## MATERIALS AND METHODS

### Cells and cell culture

The mouse thymic stromal cell line, TFGD, producing M-CSF and IL-6, was obtained from a thymoma mass that spontaneously developed in a p53-/- mouse system as described previously (4). A macrophage cell line, B6D cells, was obtained by subculturing BM-Mp in a culture medium supplemented with the culture supernatant of TFGD (50%, final concentration) for a year. The cells were cultured in Dulbecco's modified Eagle's minimum essential medium supplemented with 100 U/ml penicillin, 100 µg/ml streptomycin, and 10% heat-inactivated fetal bovine serum (Hyclone, Logan, USA).

### Cytokines and monoclonal antibodies

Recombinant human (rh) G-CSF, rhM-CSF, rhGM-CSF, rhTGF-β1 and mouse IFN-γ were purchased from PeproTech (Rocky Hill, NJ). The monoclonal antibodies (mAbs) recognizing murine cell surface markers, anti-CD11c (clone HL3) and anti-CD69 (clone H1.2F3) were purchased from Pharmingen (San Diego, CA). Anti-Dec-205 and anti-CSF1R were provided by Dr. K. Komschlies and Dr. J. Keller (National Cancer Institutes, Frederick, MD), respectively.

### Generation of BM-Mp

BM cells were isolated from femurs of C57BL/6 mice, cultured in a medium containing the culture supernatant of TFGD cells (50%, final concentration) in a 100 mm petri dish overnight. Non-adherent cells were collected after gentle shaking, counted, adjusted to 2×10^5^ cells/ml with the same medium, distributed in each well of a 6-well tissue culture plate (5 ml/well), and then incubated for 3 more days. At days 4 and 6 from the initiation of culture, non-adherent cells were removed after vigorous shaking, and then 5 ml of the same medium was added to the culture. After 8 days of culture, the adherent cells were harvested by gentle pipetting with ice-cold phosphate buffered saline (PBS) containing 0.5 mM ethylenediaminetetraacetic acid (EDTA).

### Assessment of cell proliferation (XTT assay)

XTT (Sodium 3'-[1-(phenylaminocarbonyl)-3,4-tetrazolium]-bis (4-methoxy-6-nitro) benzene sulfonic acid hydrate) (Sigma-Aldrich) was dissolved in PBS (1 mg/ml), and stored at 4℃. XTT working solution was prepared just prior to use by mixing 1 ml of XTT stock solution with 5 µl of PMS (N-methyl dibenzo-pyrazine methyl sulfate, 5 mM in PBS) (Sigma-Aldrich). The XTT working solution was added to wells of cell culture (50 µl/well), incubated at 37℃ for 4 h, and the absorbance was measured using an ELISA plate reader at 460 nm (DynaTech MR5000).

### Phenotypic analysis

Cells were stained with monoclonal antibodies recognizing murine cell surface markers as described previously (4), and flow cytometric analysis was performed using a FACSCalibur (Becton-Dickinson). Dead cells were gated out by their low forward angle light scatter intensity. In most analysis, 10,000 cells were scored.

### Measurement of APC function

The APC function of macrophages was determined by testing their ability to stimulate proliferation of anti-CD3 mAb-primed syngeneic T cells, as described previously (4). Briefly, purified T cells (1×10^6^ cells/ml) were mixed with anti-CD3 mAb (50 ng/ml; PharMingen), and then 100 µl of the cell suspension was added to each well of 96-well plates. Macrophages were treated with mitomycin-C (Sigma) for 20 minutes at 37℃, washed, and then indicated amounts of the cells were added to each well. DNA synthesis was measured by [^3^H]-thymidine (DuPont Pharmaceuticals, Wilmington, DE) incorporation (0.5 µCi/well) for the final 8 h of the 3-day culture period.

### Microarray analysis

RNA was isolated from BM-Mp and B6D cells using an RNA extraction system (RNeasy: Qiagen, Valencia, CA). Preparation of probes and hybridization processes were performed essentially as previously described (16) Briefly, cDNAs were synthesized from total RNAs by random-primed reverse transcription in the presence of Cy3-UTP or Cy5-UTP. The full-length enriched mouse cDNA microarrays were hybridized with labeled cDNA probes overnight at 65℃, and then washed in 2×SSC/0.1% SDS, washed in 1×SSC, and finally washed in 0.1×SSC. Then, these slides were scanned on a Scan Array 5000 confocal laser scanner, and the images were analyzed using ImaGene (BioDiscovery).

### Real-time PCR

One microgram of RNA isolated from BM-Mp and B6D cells was used to prepare cDNA with a TaqMan Reverse Transcription kit (Applied Biosystems, Branchburg, NJ). One microliter of each cDNA sample was then used for quantification using the SYBR Green PCR master mix (Applied Biosystems) and reactions were run on the ABI Prism 7700 Sequence Detector (Applied Biosystems). The results were normalized to GAPDH using the Quantum RNA universal 18S (Ambion, Austin, TX) and were also used to determine relative quantities. The probe, TGCACAGCTCACGGCACCGG, was labeled at the 5' end with 6-carboxyfluorescein (6-FAM) and contained the quencher dye 6-carboxy-*N,N,N',N'*-tetramethylrhodamine (TAMRA) at the 3' end. The forward primer was 5'-TGGAAAGGGCCCAGCAC-3', and the reverse primer was 5'-GCAATAGTTGGTATCCAGGGCT-3'. The relative level of TGF-β1 mRNAs was calculated as described by Liu and Saint (17).

## RESULTS

### Establishment and characterization of a macrophage cell line, B6D

**Figure 1:**
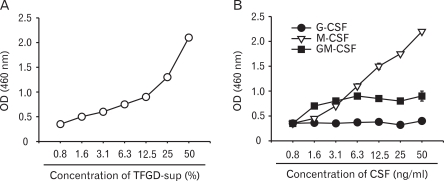
Effects of various cytokines on the growth of B6D cells. B6D cells were cultured in the presence of the indicated amounts of the culture supernatant of TFGD cells (A) or with the indicated cytokines (B) for 3 days. The proliferation of B6D cells was determined by an XTT assay. The results show the mean±S.D. of three independent experiments.

**Figure 2:**
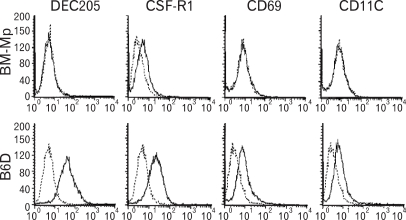
Phenotypic differences between BM-Mp and B6D cells. The cells were stained with the indicated mAbs, washed and analyzed by flow cytometry. Levels of expression (thin line) were illustrated in comparison to isotype control (dotted line).

BM-Mp, which was generated from BM cells of C57BL/6 mice by culturing in a medium containing the culture supernatant of TFGD cells (50%, final concentration), was continuously subcultured in a medium supplemented with the culture supernatant of TFGD. After a year of subculture, the macrophages, named B6D, exhibited M-CSF-dependency as well as TFGD-supernatant-dependency in the growth pattern (Fig. 1). Flow cytometric analysis showed that B6D cells expressed CD11b, CD24, CD44, CD45, CD54, MHC class II (I-A^b^), CD80, CD86 and CD40 to a level similar to that of BM-Mp which was freshly generated from BM cells using TFGD-supernatant (data not shown). However, the expression levels of DEC205, CSF-R1, CD69 and CD11c were significantly higher in B6D cells compared to that of freshly generated BM-Mp (Fig. 2).

### Comparison of the APC function of B6D cells and BM-Mp

**Figure 3:**
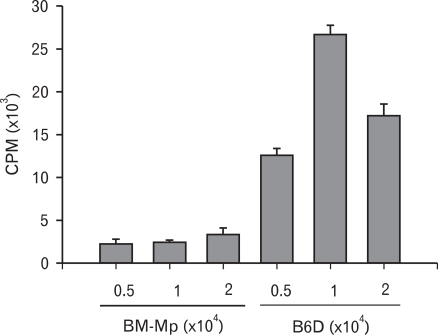
Comparison of the APC functions of BM-Mp and B6D cells. Syngeneic anti-CD3 mAb-primed T cells (1×10^5^ cells/well) were cultured with the indicated number of BM-Mp or B6D cells. Proliferation of T cells was measured by [^3^H]-thymidine incorporation for the final 8 h of the culture period of 3 days. The results show the mean±S.D. of three independent experiments.

The APC function of B6D cells and BM-Mp was comparatively studied by testing their ability to induce proliferation of syngeneic anti-CD3 mAb-primed T cells. Consistent with previous observations (4), BM-Mp cells were defective in inducing proliferation of syngeneic anti-CD3 mAb-primed T cells. However, B6D cells efficiently enhanced the proliferation of syngeneic anti-CD3 mAb-primed T cells (Fig. 3). The proliferation-inducing activity of B6D cells was most potent when the ratio of B6D cells and syngeneic anti-CD3 mAb-primed T cells was 1:10.

### Microarray analysis of the differential gene expression between B6D cells and BM-Mp

To examine any differences in gene expression between B6D cells and BM-Mp, mRNAs were isolated from each cell type, and then cDNAs were synthesized from total mRNAs by random-primed reverse transcription in the presence of Cy3-UTP (green color) or Cy5-UTP (red color). The microanalysis plate contained 1,200 genes. To confirm the differential expression of genes, microarray analysis was repeated with the cDNAs reversely labeled with the dyes. Microarray analysis identified approximately 89 genes that were differentially expressed (data not shown). The genes that were profoundly different in expression are shown in Table I. Among these, TGF-β1, which was highly expressed in BM-Mp compared to B6D cell, was selected for further experiments, because TGF-β1 has been known to inhibit immune responses in a variety of systems (11-15).

### Effects of blocking or addition of TGF-β1 on the APC function of macrophages

**Figure 4:**
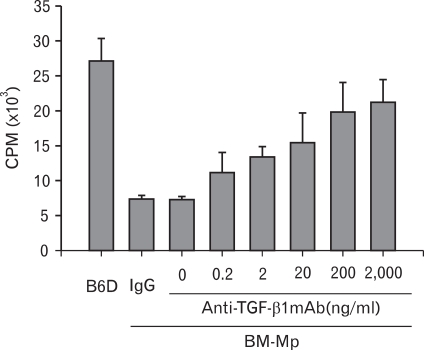
Recovery of APC function of BM-Mp by blocking with anti-TGF-β1 mAb. Syngeneic anti-CD3 mAb-primed T cells (1×10^5^ cells/well) were cultured with BM-Mp (1×10^4^ cells/well) in the presence of the indicated amounts of anti-TGF-β1 mAb. IgG is an isotype control for anti-TGF-β1 mAb. The results show the mean±S.D. of three independent experiments.

**Figure 5:**
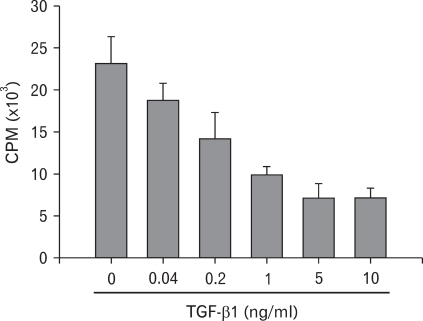
Inhibition of the APC function of B6D cells by addition of TGF-β1. Syngeneic anti-CD3 mAb-primed T cells (1×10^5^ cells/well) were culture with B6D cells (1×10^4^ cells/well) in the presence of the indicated amounts of rhTGF-β1. The results show the mean±S.D. of three independent experiments.

To confirm that TGF-β1 produced from BM-Mp was responsible for the defective APC function of BM-Mp, two experiments, blocking of TGF-β1 with anti-TGF-β1 mAbs and addition of TGF-β1, were performed. As shown in Fig. 4, addition of anti-TGF-β1 mAbs to mixed cultures of BM-Mp and anti-CD3 mAb-primed syngeneic T cells dose-dependently increased the proliferation of T cells. Conversely, addition of TGF-β1 to mixed cultures of B6D cells and anti-CD3 mAb-primed syngeneic T cells dose-dependently inhibited the proliferation of T cells (Fig. 5). These results indicated that TGF-β1 produced from BM-Mp was responsible for the defective APC function of BM-Mp.

### Quantitative comparison of the expression of TGF-β1 mRNA

In order to compare the expression of TGF-β1 mRNA more accurately, real time PCR analysis was performed with the cDNAs obtained from BM-Mp and B6D cells. The quantity of mRNA was compared by the values of 2^-ΔΔCT^ (relative level). As shown in Table II, BM-Mp expressed a 2.8-fold higher level of TGF-β1 mRNA compared to B6D cells under unstimulated conditions. The ratio of difference in expression of TGF-β1 mRNA was not significantly altered after stimulation with LPS (100 ng/ml), or IFN-γ (100 U/ml). The variability between triplicate assays of the same cDNA sample was typically less than 5%.

## DISCUSSION

The present study investigated the possible cause of defective APC functioning of macrophages that were generated *in vitro* using M-CSF. Macrophages are usually strongly phagocytic for IgG-opsonized sheep red blood cells (SRBCs), produce nitric oxide (NO) in response to interferon IFN-γ plus LPS, and express cell surface molecules that are known to be associated with mouse macrophages (2-4). However, macrophages generated from BM cells *in vitro* using M-CSF alone or in combination with IL-6 are defective in APC functions. Macrophages generated from CD34+ progenitors by cytokines produced from a renal carcinoma cell line were also shown to be defective in APC function, although they exerted powerful phagocytic activity and expressed the same surface phenotype markers with peripheral blood macrophages (18). The renal carcinoma cell line was shown to produce M-CSF and IL-6 (18). This feature is unique, because DCs generated from BM cells or peripheral blood monocytes with GM-CSF exert strong APC functions (19,20).

To address the possible reasons for defective APC functioning of the macrophages generated *in vitro* using M-CSF, we compared differences in gene expression between a macrophage cell line B6D, which exerted efficient APC function, and that of macrophages generated from BM cells with M-CSF and IL-6. Microarray analysis showed that TGF-β1 was highly expressed in macrophages generated from BM cells with M-CSF, compared to B6D cells. Blocking experiments with anti-TGF-β1 mAbs as well as additional experiments with TGF-β1 confirmed that TGF-β1 produced by macrophages that were generated from BM cells *in vitro* using M-CSF was responsible for the defective APC function. Real time PCR analysis also confirmed that TGF-β1 was highly expressed in the macrophages generated by BM cells *in vitro* using M-CSF and IL-6.

Macrophages can be generated from CD34+ progenitors *in vitro* using M-CSF, which is a hematopoietic glycoprotein that stimulates the proliferation and differentiation of BM progenitor cells into myeloid cells. M-CSF plays an important role in monocyte/macrophage homeostasis (21,22). M-CSF, however, by itself is not effective in inducing macrophage differentiation from BM progenitors (1,18). Earlier studies showed that M-CSF synergies with IL-6 in the generation of macrophages from BM progenitor cells (33,34,38). We used the culture supernatant of TFGD cells, which was shown to contain large amounts of M-CSF and IL-6 (4), to produce macrophages from BM progenitor cells *in vitro*. The macrophages generated by the culture supernatant of TFGD cells were defective in APC function, as shown by the present study as well as by an earlier study (4).

One of the most potent activities of TGF-β1 on lymphocytes is its anti-proliferative effect. TGF-β1 inhibits the proliferation of T lymphocytes and B-lymphocytes, thymocytes, large granular lymphocytes, and NK cells (11-15). Studies using peripheral blood mononuclear cells, monocytes and T lymphocytes suggest that TGF-β1 may function as a strong inhibitor of the expression of many cytokines involved in the effector functions of activated cells (11,15). TGF-β1 was shown to inhibit the effects and/or the production of IFN-γ, TNF-α, TNF-β, IL-1, IL-2 and IL-3, as well as the expression of IL-2 receptor (22-25). Thus, the inhibition of cytokine activity is presumably a major factor in TGF-β1-induced immunosuppression. Further evidence of the strong immunosuppressive effect of TGF-β1 on lymphocytes is the reported downregulation of IFN-γ-induced MHC class II antigen expression by TGF-β1 in both lymphoid and non-lymphoid cells (26). Taken together, the present study demonstrated that TGF-β1 produced from the M-CSF-generated macrophages was responsible for the defective APC functioning of the macrophages.

## Figures and Tables

**Table I:**
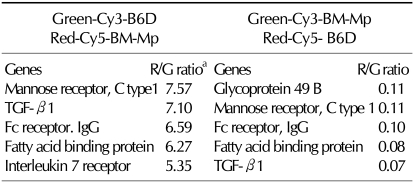
Microarray analysis for the differential expression of genes between B6D cells and BM-Mp

^a^R/G ratio indicates the fluorescence ratio of red (Cy5) vs green (Cy3).

**Table II:**
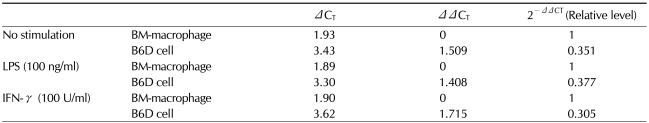
Expression of TGF-β1 mRNA

RNA samples were isolated from BM-Mp and B6D cells that were stimulated with LPS or IFN-γ for 48 h, and then used to generate the corresponding cDNA samples. Real time PCR reactions were run on an ABI Prism 7700 Sequence Detector (Applied Biosystems) with the probes described in the Methods section. Relative gene expression levels were calculated as described by Liu and Saint (17).
